# Mental health and social support among Royal Canadian Mounted Police cadets

**DOI:** 10.3389/fpsyg.2023.1092334

**Published:** 2023-02-13

**Authors:** Jolan Nisbet, Laleh Jamshidi, Katie L. Andrews, Sherry H. Stewart, Robyn E. Shields, Taylor A. Teckchandani, Kirby Q. Maguire, R. Nicholas Carleton

**Affiliations:** ^1^Canadian Institute for Public Safety Research and Treatment, University of Regina, Regina, SK, Canada; ^2^Department of Psychiatry, Dalhousie University, Halifax, NS, Canada; ^3^Department of Psychology and Neuroscience, Dalhousie University, Halifax, NS, Canada; ^4^Department of Community Health and Epidemiology, Dalhousie University, Halifax, NS, Canada; ^5^Anxiety and Illness Behavior Laboratory, Department of Psychology, University of Regina, Regina, SK, Canada

**Keywords:** mental health, social support, occupational health, public safety personnel, RCMP cadets

## Abstract

**Introduction:**

Certain populations, such as public safety personnel (PSP), experience frequent exposures to potentially psychologically traumatic events and other occupational stressors, increasing their risk for mental health challenges. Social support has been evidenced as a protective factor for mental health. However, research examining perceived social support and its associations with symptoms related to mental disorders among PSP recruits is limited.

**Methods:**

RCMP cadets (*n* = 765, 72% male) completed self-report surveys assessing: sociodemographic information, social support, and symptoms related to posttraumatic stress disorder, major depressive disorder, generalized anxiety disorder, social anxiety disorder, panic disorder, and alcohol use disorder.

**Results:**

The results indicated statistically significant associations between higher social support and decreased odds of positive screens for generalized anxiety disorder, social anxiety disorder, and panic disorder (i.e., significant Adjusted Odds Ratios = 0.90 to 0.95).

**Discussion:**

Cadets’ perceived levels of social support are comparable to the Canadian general population and higher than serving RCMP. Social support appears to offer a protective element against anxiety-related disorders among participating cadets. Reductions in perceived levels of social support may be a function of RCMP service. Factors contributing to decreased levels of perceived social support should be considered.

## Introduction

Social support is a multifaceted construct, which can be conceptualized as “a social network’s provision of psychological and material resources intended to benefit an individual’s ability to cope with stress” (Cohen, 2004, p: 676). Notably, *intentions* to help others cope with stress may not always align with *perceptions* of the support received. Perceptions of psychological and material resources associated with social support can be categorized into three groups: (1) informational (e.g., giving advice, providing guidance); (2) instrumental (e.g., providing material or financial resources, helping with routine tasks); and (3) emotional (e.g., expressing empathy, reassuring, providing the opportunity to process emotions; House et al., 1985; Vig et al., 2020). Social support may be provided by spouses, partners, family, friends, co-workers, or professionals (Cohen and Wills, 1985). The extent of social support an individual perceives may vary depending on their depth of integration within a relationship or organization (Cohen and Wills, 1985). High levels of perceived social support can provide direct effects in improving mental and physical health, and can buffer against the adverse effects of stress on health (Uchino et al., 1996; Cohen et al., 2000; Patterson, 2003; Southwick et al., 2005; Ozbay et al., 2007; Thoits, 2011; Santini et al., 2015; Hansson et al., 2017; Vig et al., 2020; Nero et al., 2022).

Social support appears particularly relevant for occupations involving high-stress levels, physical exertion, and exposure to distressing events. Public safety personnel (PSP) are routinely exposed to organizational stressors (e.g., staff shortages, lack of appropriate resources, inconsistent approaches to leadership), operational stressors (e.g., fatigue, shift work, job-related injuries), and diverse potentially psychologically traumatic events (PPTEs; e.g., life-threatening natural disaster, sudden violent death, serious transportation accident; McCreary and Thompson, 2006; Carleton et al., 2018a). Associations between PPTEs and higher prevalence of mental health disorders have been reported among a large sample of Canadian PSP (Carleton et al., 2018b), thus repeated exposure to PPTEs may be, at least in part, one explanation for a higher prevalence of positive screens for mental health disorders among PSP (Carleton et al., 2018b). Conversely, increased social support among various PSP sectors, where there are high levels of stressor and trauma exposure, has been associated with the buffering of the effects of stress on mental health, such as decreased symptoms of major depressive disorder (MDD) and posttraumatic stress disorder (PTSD) among various PSP sectors (Vig et al., 2020). Nonetheless, PSP report experiencing diminishing social support as a function of their vocational service perhaps due to occupational stressors and/or PPTEs (Regehr et al., 2003; Baek et al., 2022). Moreover, sociodemographic variables of gender and marital status may impact how social support is perceived. Gender appears to influence how PSP experience social support, as women PSP emphasize that social support is more reciprocal in nature than their colleagues (Kaur et al., 2021). PSP who are married or in a common-law relationship report that their partners are their first point of contact when seeking mental health support (Carleton et al., 2020).

Research on perceived social support and associations with mental health challenges *before* exposure to diverse occupational stressors and various PPTEs among PSP recruits is limited (Regehr et al., 2003). Expanding research at the recruit level can provide a benchmark to analyze fluctuations in perceived social support at various career stages. The current paper begins to address these gaps by considering perceived social support among newly recruited Royal Canadian Mounted Police (RCMP) cadets at the start (i.e., pre-training stage, T1) of the Cadet Training Program (CTP; Carleton et al., 2022). The CTP is a nationwide training program in Canada and one of the largest training programs for Canadian PSP. The CTP includes a variety of stressors; consequently, high levels of perceived social support may help cadets to navigate through various challenges during the 26-week program. Notably, cadets must undergo: (1) a wide range of testing (e.g., a full medical exam, lab tests, physical stamina, psychological examinations); (2) operating in para-military conditions; (3) relocating to Depot in Regina, Saskatchewan; (4) adjusting to living conditions at Depot; (5) forming new relationships with facilitators and other cadets; and (6) balancing CTP obligations with prior relationships.

Data for the current paper are derived from the RCMP Study, a large-scale longitudinal evaluation of RCMP cadets’ mental health, with details available in a published Protocol Paper (Carleton et al., 2022). The RCMP Study data collection provides an opportunity to address several gaps in the literature regarding newly recruited RCMP cadets. The current paper was designed to assess perceptions of social support among RCMP cadets starting the CTP by: (1) examining perceived social support among RCMP cadets and assessing for differences across sociodemographic characteristics (i.e., gender, sex, age, ethnicity, marital status, province of residence, education); (2) comparing the cadets’ self-reported levels of social support with those in the Canadian general population; (3) comparing the cadets’ self-reported levels of social support with levels reported by serving RCMP; and (4) examining associations between social support and positive screens for several mental health disorders.

RCMP cadets starting the CTP were expected to report different levels of perceived social support based on: (1) marital status (e.g., cadets who are married or in common-law relationships were expected to report higher levels of social support than single, widowed, separated, or divorced cadets; Carleton et al., 2020; Nero et al., 2022); (2) gender and sex dynamics (e.g., female cadets were expected to report higher levels of social support than male cadets; Bellman et al., 2003); (3) comparisons with the Canadian general population. Directional predictions were not made for this hypothesis since cadets might report higher levels of perceived social support than members of the Canadian general population as a function of self-selection biases related to meeting the rigorous CTP screening criteria (Carleton et al., 2022) or might report lower levels of social support due to high levels of factors such as self-reliance; and (4) comparisons with RCMP. Cadets were expected to report higher levels of perceived social support than serving RCMP (i.e., serving members were expected to report lower levels of social support as a function of the diminishing social support associated with service; Regehr et al., 2003). Additionally, inverse relationships between perceived social support and self-reported mental health disorder symptoms associated with Panic Disorder (PD), Generalized Anxiety Disorder (GAD), Social Anxiety Disorder (SAD), PTSD, MDD, and Alcohol Use Disorder (AUD) were expected (Ozbay et al., 2007; Vig et al., 2020).

## Methods

### Procedure

Data for the current paper were collected as a part of the broader RCMP Study. The associated protocol paper provides full details of the RCMP longitudinal Study (Carleton et al., 2022). The RCMP Study was approved by the University of Regina Ethics Board on April 10, 2019 (File #2019–055) and by the RCMP Research Ethics Board on April 12, 2019 (File #SKM_C30818021312580). The project is bound by the *Privacy Act*, R.S., 1985, c. P-21 and the Personal Information Protection and Electronic Documents Act, SC. 2000, c.5. The RCMP Study was also approved through a Privacy Impact Assessment as part of the overall approval by the National Administrative Records Management System (NARMS; File #201611123286) and Public Services and Procurement Canada (PSPC; File #201701491/M7594174191).^1^

### Data and sample

The current sample consisted of RCMP cadets at the start (i.e., pre-training stage, T1) of the CTP. To qualify for participation in the CTP, an individual had to be a Canadian citizen or permanent resident, 19 to 57 years old, and able to fluently read, write, and speak English or French (Hembroff and Krätzig, 2020). Cadets also had to meet several additional CTP recruiting requirements, including security clearances, medical examinations, a polygraph test, and minimum physical standards (Hembroff and Krätzig, 2020). There were no conditions excluding participation in the RCMP Study for persons otherwise qualified for the CTP.

Upon arrival at Depot, cadets were invited to attend a recruitment session delivered by research team members. The session included: (1) video content from serving RCMP members (~10 min); (2) introductions to the research team; (3) a didactic lecture with a slide show presentation (~35 min long); and (4) an opportunity for potential participants to ask questions (~15 min). The presentation outlined the RCMP Study rationale, design, requirements, expected outcomes, and potential benefits to the RCMP, the broader PSP community, and all Canadians.

Cadets who agreed to participate were then asked to attend an on-boarding session which involved the formal consent process and the subsequent completion of a full assessment survey. Data were collected through self-report questionnaires, which included sociodemographic information, symptom measures, and a measure of perceived social support. The current data were collected at the pre-training stage of the CTP as part of the first full assessment of cadets within the RCMP Study. All Study-related activities were completed during the cadets’ training time. As a precaution of the COVID-19 pandemic, RCMP Depot was closed from March 2020 to October 2020. All research-related activities (i.e., recruitment, onboarding, clinical interviews) were conducted remotely from October 2020 to June 2022. A total of (*n =* 1, 696) cadets were invited to participate in the Study and a total of (*n =* 890) agreed to participate. Analyses for the current paper were limited to a sample of those cadets who completed the survey measure assessing perceived social support (*n* = 765) which represented 86% of those cadets who were recruited into the Study.

### Perceived social support

The Social Provisions Scale (i.e., SPS-10) was used to assess perceived social support (Caron, 2013). Participants indicated the extent of social support they perceived receiving across 10-items. Each item was rated using a four-point Likert scale ranging from 1 (i.e., *strongly disagree*) to 4 (i.e., *strongly agree*) and scored continuously (Orpana et al., 2019). Total scores of 30 can be interpreted as a respondent indicating high levels of perceived social support (Orpana et al., 2019). Evaluation of the SPS-10 has demonstrated excellent internal consistency for the global scale (α = 0.880), with the alphas for the six subscales (i.e., attachment, social integration, reassurance of worth, sense of reliable alliance, guidance, and the opportunity for nurturance) ranging from 0.528 to 0.900, and strong convergent validity with the original 24-item scale (*r* = 0.930; Caron, 2013).

### Self-report symptom measures

All mental health symptoms were self-reported using the screening measures described below. A ‘positive screen’ on any of the following measures indicated that the individual has self-reported symptoms consistent with persons who have been diagnosed with a particular mental health disorder, which is not necessarily synonymous with meeting diagnostic criteria themselves. Individuals who completed the self-report measures and screened positive would require the evaluation of a trained clinician to diagnose a specific mental health disorder.

The Panic Disorders Symptoms Severity Scale (PDSS-SR) was used to assess symptoms related to panic disorder (PD; Shear et al., 1997; Houck et al., 2002). Participants first read the definition of a panic attack, and the accompanying symptoms. From the accompanying symptoms, at least four had to be endorsed (e.g., rapid or pounding heartbeat, sweating, nausea, feeling of choking) for a panic attack to have occurred. If the participant reported having ever experienced a panic attack, or experiencing a panic attack in the past week, they were asked additional questions rated on a five-point Likert scale (i.e., 0 = *none* to 4 = *extreme*). Participants who were never administered the PDSS-SR due to never experiencing a panic attack were considered to have screened negative for PD. A positive screen for PD required the PDSS-SR total score to be seven or greater, which can be used to identify persons with clinically significant anxiety and distress (Shear et al., 2001; Houck et al., 2002). The self-report version has demonstrated excellent psychometrics with a strong internal consistency (α = 0.92) and an intraclass correlation coefficient of 0.81 (Houck et al., 2002).

The Generalized Anxiety Disorder Scale (GAD-7) was used to assess generalized anxiety disorder (GAD) symptoms (Spitzer et al., 2006; Beard and Björgvinsson, 2014). Participants indicated the extent to which seven symptoms of anxiety bothered them in the previous 2 weeks. Ratings were made on a four-point Likert Scale (i.e., 0 = *not at all* to 3 = *nearly every day*; Spitzer et al., 2006). A positive screen for GAD required a total score of greater than nine (Swinson, 2006). The GAD-7 has shown good reliability, and construct, criterion, procedural, and factorial validity (Spitzer et al., 2006) as well as good internal consistency (α = 0.89) and inter-item correlations 0.45–0.65 in a community sample (Löwe et al., 2008).

The Social Interaction Phobia Scale (SIPS) is a 14-item measure that was used to assess social anxiety disorder (SAD) symptoms (Carleton et al., 2009). The SIPS includes three subscales to assess social interaction anxiety, fear of overt evaluation, and fear of attracting attention, respectively. Each item is rating on a five-point Likert Scale (i.e., 0 = *not at all characteristic of me*, 4 = *entirely characteristic of me*). There is no specific time-window used. A positive screen for SAD requires a SIPS total score of 20 or greater (Carleton et al., 2009). The SIPS has demonstrated overall excellent internal consistency (α = 0.92), convergent and discriminant validity in a large and independent sample (Reilly et al., 2012).

The PTSD Checklist for DSM-5 (PCL-5) was used to assess PTSD symptoms (Blevins et al., 2015). Participants rated how bothered they had been by 20 common symptoms of PTSD in the past month on a five-point Likert scale, from 0 (i.e., *not at all*) to 4 (i.e., *extremely*). Positive screens on the PCL-5 are determined by meeting the minimum DSM-5 criteria for each PTSD cluster (i.e., avoidance, hyperarousal, intrusions, and mood and cognitive changes) and exceeding the minimum clinical cut-off of greater than 32 on the total score (Weathers et al., 2013). Psychometric evaluation of the PCL-5 has demonstrated strong internal consistency (α = 0.94) and good test–retest reliability (r = 0.82) within populations exposed to PPTEs (Blevins et al., 2015). The PCL-5 has a strong convergent validity with other trauma measures (Weathers et al., 1994).

The Patient Health Questionnaire (PHQ-9) is a 9-item self-report questionnaire that was used to assess MDD symptoms (Kroenke et al., 2001). Participants indicated how bothered they had been by depressive symptoms in the past 2 weeks by responding to each item using a four-point Likert scale (i.e., 0 = *not at all* to 3 = *nearly every day*; Manea et al., 2015). A positive screen for MDD is indicated by a score of nine or greater (Manea et al., 2015). Psychometric support for the PHQ-9 details a sensitivity of 88% and specificity of 88% (Löwe et al., 2004a, 2004b). Psychometric evaluation of the PHQ-9 has demonstrated good internal consistency (α = 0.89) and test–retest reliability (*r* = 0.84) within the general population (Kroenke et al., 2001).

The Alcohol Use Disorders Identification Test (AUDIT) is a 10-item self-report questionnaire to assess alcohol consumption and dependence over the past 12 months (Saunders et al., 1993; World Health Organisation AUDIT, 2001; Gache et al., 2005). Participants were asked questions about their drinking behaviors and negative alcohol-related consequences. Ratings were made using Likert scales that varied across items. A positive screen for AUD required the total AUDIT score to be 15 or higher (Babor et al., 2001; Gache et al., 2005). Psychometric evaluation of the AUDIT has demonstrated good internal consistency (α = 0.81), good test–retest reliability (*r* = 0.83 to 0.95) within the general population, and (α = 0.81) in a police-specific population (Daeppen et al., 2000; Davey et al., 2000; Reinert and Allen, 2007). Criterion validity for correlations between AUDIT and four dimensions of alcohol consumption ranged from 0.47 to 0.66 and AUDIT questions were moderately sensitive (i.e., 54 to 79%) for criteria corresponding to heavy drinking (Bradley et al., 1998).

## Statistical analyses

SPSS v.28 Premium (IBM, 2021 New York, United States) was used to conduct the quantitative analyses. Participants were grouped into sociodemographic categories for comparisons (i.e., gender, sex, age, ethnicity, marital status, former province of residence, education). First, the descriptive analyses provided information about the frequencies and percentages of participant sociodemographic variables. Means and the standard deviations of SPS-10 scores were calculated across different sociodemographic categories. A series of independent sample *t*-tests and one-way analyses of variance tests (ANOVA) were used to assess for differences in SPS-10 scores across sociodemographic categories, with Holm-Bonferroni adjustments applied to alpha levels in post-hoc analyses to control Type I errors in multiple comparisons. The item-level SPS-10 responses from cadets were compared to data from the Canadian general population (Statistics Canada, 2013; Orpana et al., 2019) and serving RCMP using a series of one-sample *t*-tests and independent sample *t*-tests. ^2^ The alpha level was set to 0.05 for all analyses. Second, a series of logistic regressions were conducted to assess the likelihood of screening positive for PD, GAD, SIPS, PTSD, MDD and AUD self-report measures based on SPS-10 scores.

## Results

Participant sociodemographic details are provided in Table 1. Most participants were men (72.2%) and self-identified as male (72.0%). Most participants were between the age of 19 and 29 (60%), single (47.1%), or married/in a common-law relationship (43%). Participants were mainly White/Caucasian (79.0%) and from Western Canada (52.9%; i.e., British Columbia, Alberta, Saskatchewan, or Manitoba). Most participants reported having some post-secondary school (43.1%).

**Table 1:** Frequencies for sociodemographic variables and mean social support scores among RCMP Cadets.

Sociodemographic variables	*% (n)*	SPS-10 scores	*Effect size*
		*M (SD)*	
**Gender**			
Man	72.2 (552)	35.90 (4.45)	*d*^1^ = 0.049
Woman	24.5 (188)	36.13 (5.62)	
Non-binary	^	^	
Transgender	^	^	
Two-spirited	^	^	
**Sex**			
Male	72.0 (551)	35.90 (4.45)	*d*^1^ = 0.042
Female	25.1 (192)	36.10 (5.60)	
**Age (years)**			
19–29	60.0 (459)	36.17 (4.53)	$\eta_{p}^{2}$^2^= 0.009
30–39	28.0 (214)	35.30 (5.40)	
40–49	6.1 (47)	36.70 (3.88)	
50–59	0.7 (5)	34.20 (3.96)	
Other/No response	5.2 (40)	–	
**Ethnicity**			
Asian	6.4 (45)	34.71 (5.74)	$\eta_{p}^{2}$^2^= 0.008
Black	3.4 (24)	35.67 (4.71)	
First Nations/Inuit/Metis	3.3 (23)	35.96 (4.13)	
Hispanic	1.6 (11)	37.27 (3.52)	
South Asian	6.4 (45)	35.64 (5.83)	
White/Caucasian	79.0 (558)	36.16 (4.45)	
**Marital Status**			
Single	47.1 (360)	35.72 (4.81)	$\eta_{p}^{2}$^2^= 0.002
Separated/Divorced/Widowed	1.4 (11)	34.91 (4.21)	
Married/Common-Law	43.0 (329)	36.05 (4.89)	
Other/No response	8.5 (65)	–	
**Province of residence**			
Western Canada (BC, AB, SK, MB)	52.9 (405)	35.77 (5.06)	$\eta_{p}^{2}$^2^= 0.003
Eastern Canada (ON, QC)	34.5 (264)	36.21 (4.49)	
Atlantic Canada (PEI, NS, NB, NFL)	11.2 (86)	36.50 (3.92)	
Northern Territories (YK, NWT, NVT)	1.1 (8)	36.25 (4.37)	
Other/No response	^	–	
**Education**			
High school graduate or less	10.2 (78)	35.37 (4.98)	$\eta_{p}^{2}$^2^= 0.004
Some post-secondary school	43.1 (330)	35.81 (4.83)	
University degree/4-year college or higher	39.7 (304)	36.25 (4.72)	
Other/No response	7.0 (53)	–	
**Total sample**	100 (765)	36.01 (4.74)	–

*p ≤ 0.05; **p ≤ 0.01; ***p ≤ 0.001. There are no significant differences across demographic groups in social support scores. ^The sample size is between 1 and 4, so the exact data cannot be presented.

1. Cohen’s *d*.

2. Partial eta squared.

- No data reported.

High levels of social support (i.e., scores of 30 or greater) were reported across sociodemographic categories but no statistically significant differences were observed. Female cadets reported slightly higher scores (36.10 ± 5.60) than male cadets (35.90 ± 4.45). Cadets from 40 to 49 years of age reported the highest level of social support (36.70 ± 3.88), followed by cadets from 19 to 29 years of age (36.17 ± 4.53). Cadets who identified as Hispanic reported the highest levels of social support (37.27 ± 3.52), followed by those identifying as White/Caucasian (36.16 ± 4.45). Cadets who are married or in a common-law relationship reported the highest level of social support (36.05 ± 4.89), followed by single cadets (35.72 ± 4.81), and separated or divorced cadets (34.91 ± 4.21). Cadets from Atlantic Canada reported the highest level of social support (36.50 ± 3.92). Cadets with a university degree, 4-year College or higher level of education reported the highest level of social support (36.25 ± 4.72). Although there were no statistically significant results, the scores illustrate the high levels of perceived social support across sociodemographic categories and provide greater details on the perceived social support among cadets.

Associations between SPS-10 total scores and positive screens on self-report mental health symptom measures are provided in Table 2. The comparisons are presented as odds ratios (ORs), which measure the association between self-reported social support (i.e., SPS-10) and mental health disorder symptoms (i.e., PD, GAD, SAD, PTSD, MDD, AUD). Associations were also assessed after controlling for sociodemographic covariates (i.e., sex, age, ethnicity, marital status, province of residence, and education) as adjusted odds ratios (AORs). ORs and AORs both showed-statistically significant inverse associations between SPS-10 scores and odds of screening positive for GAD, SAD, and PD (ORs ranged from 0.88 to 0.95;AORs ranged from 0.90 to 0.95). Cadets were less likely to screen positive for these mental disorders as the level of social support increases. No statistically significant associations were observed between perceived social support and symptoms of PTSD, or MDD. Odds ratios could not be calculated assessing the association between social support and AUD as no cadets screened positive for AUD-based on an AUDIT score of 15 or higher.

**Table 2:** Relationship between social support scores and positive screens for mental health disorders.

	OR [95% CI]	AOR [95% CI]
Posttraumatic stress disorder (PCL-5)	0.97 [0.89, 1.07]	0.99 [0.90, 1.09]
Major depressive disorder (PHQ-9)	0.98 [0.92,1.04]	0.99 [0.94, 1.05]
Generalized anxiety disorder (GAD-7)	0.95* [0.91, 0.99]	0.95* [0.90, 0.99]
Social anxiety disorder (SIPS)	0.93* [0.88, 0.99]	0.93* [0.87, 0.99]
Panic disorder (PDSS-SR)	0.88** [0.82, 0.95]	0.90* [0.83, 0.98]
Alcohol use disorder (AUDIT)	–	–

AOR = Odds ratio adjusted for sex, age, ethnicity, marital status, province of residence, and education; PCL-5 = PTSD Checklist for DSM-V; PHQ-9 = Patient Health Questionnaire-9; GAD-7 = General Anxiety Disorder-7; SIPS = Social Interaction Phobia Scale; AUDIT = Alcohol Use Disorder Identification. Please note the Alcohol Use Disorder line is blank as no cadets met the criteria at the set cut point (i.e., 15). Test; ^*^p < 0.05, ^**^p < 0.01, ^***^p < 0.001.

At the individual-item level, cadets reported statistically significant lower scores on item-four (i.e., *I have close relationships that provide me with a sense of emotional security and well-being*; *d* = 0.100, *p* < 0.01) and cadets reported statistically significant higher scores on item nine (i.e., *There are people who admire my talents and abilities*; *d* = 0.082; *p* < 0.05) when compared to the Canadian general population (See Table 3). A statistically significant large effect size (*d =* 0.832, *p* < 0.001) was observed when comparing cadets’ total SPS-10 scores with those of serving RCMP, indicating that cadets reported statistically significant higher levels of perceived social support compared to serving RCMP. This difference is also reflected at the individual-item level, with statistically significant medium effect sizes (*d*s ranging from 0.561 to 0.783; all *p*s < 0.001) observed on all SPS-10 items with cadets scoring higher compared to serving RCMP.

**Table 3:** Comparing mean social support scores of RCMP Cadets with the Canadian Population and serving RCMP.

SPS-10 items	General Canadian population (*n* = 22,486)^3^	RCMP cadets (*n* = 765)	Comparing cadets with general population	Previously published serving RCMP (*n* = 1,214)	Comparing cadets with serving RCMP
	Mean (*SD*)	Mean (*SD*)	Effect size (Cohen’s *d*)	Mean (*SD*)	Effect size (Cohen’s *d*)
1. There are people I can depend on to help me if I really need it	3.67 (1.50)	3.67 (0.55)	0.008	3.27 (0.69)	0.618***
2. There are people who enjoy the same social activities I do	3.54 (1.50)	3.56 (0.59)	0.026	3.16 (0.63)	0.645***
3. I feel part of a group of people who share my attitudes and beliefs	3.45 (1.50)	3.48 (0.60)	0.047	2.96 (0.70)	0.783***
4. I have close relationships that provide me with a sense of emotional security and well-being	3.59 (1.50)	3.53 (0.59)	0.100**	2.99 (0.80)	0.752***
5. There is someone I could talk to about important decisions in my life	3.65 (1.50)	3.68 (0.54)	0.062	3.24 (0.75)	0.655***
6. I have relationships where my competence and skills are recognized	3.53 (1.50)	3.51 (0.60)	0.036	3.04 (0.67)	0.725***
7. There is a trustworthy person I could turn to for advice if I were having problems	3.66 (1.50)	3.67 (0.54)	0.012	3.23 (0.69)	0.679***
8. I feel a strong emotional bond with at least one other person	3.68 (1.50)	3.68 (0.55)	0.003	3.31 (0.71)	0.561***
9. There are people who admire my talents and abilities	3.49 (1.50)	3.54 (0.59)	0.082*	3.10 (0.69)	0.665***
10. There are people I can count on in an emergency	3.70 (1.50)	3.70 (0.51)	0.004	3.34 (0.65)	0.608***
Total SPS-10 score	36.04 (7.50)	36.01 (4.74)	0.007	31.65 (5.52)	0.832***

^*^p < 0.05, ^**^p < 0.01, ^***^p < 0.001.

3 The Canadian Population scores are taken from Statistics Canada (2012). The SEM was converted to SD based on the assumption that all respondents completed the questionnaire.

## Discussion

RCMP cadets reported minimal differences in levels of perceived social support across sociodemographic categories. Perceived social support was high in each category. Extant literature suggests that spouses are most likely to provide PSP with mental health support (Carleton et al., 2020; Nero et al., 2022). Almost half of the participants (i.e., 43%) in the current paper reported being married or in a common-law relationship. The cadets who reported being married or in a common-law relationship reported the highest levels of perceived social support. However, cadets who reported other marital statuses (i.e., single, divorced or separated) also reported comparatively high levels of perceived social support with no statistically significant differences detected across marital status groups. The data collected did not include the category of ‘dating’ which may be an important form of social support in younger individuals (Adamczyk and Segrin, 2015) and may help to explain the relatively high levels of social support even in the ‘single’ cadets. The current paper indicates that regardless of marital status, cadets have cultivated relationships that provide the perception of perceived social support as cadets begin the CTP. The findings support RCMP Depot continuing the Family Workshops, which are open to all family members and friends during the Graduation Program (RCMP, 2009). Future researchers may consider opportunities for virtual workshops or training sessions to spouses, common-law partners, family, and friends at various stages during the CTP which may provide continuity in social support as cadets progress through the CTP and enter into service as an RCMP member, where social support appears to degrade.

Existing literature suggests the possibility of gendered differences in social support (Bellman et al., 2003; Kaur et al., 2021). However, all cadets reported similarly high levels of perceived social support in the current paper. The current paper suggests that both women and men start the CTP with high levels of perceived social support. Additional qualitative research may provide further nuance on the gendered perceptions of social support among cadets; for example, changing social interpretations and mores may be a factor. Male cadets may feel less stigma to lean on peers, or other support systems than in the past due to various efforts within the RCMP and wider Canadian society. Future research may consider the extent to which social support is reciprocal based on gender-dynamics, which would provide an additional layer of understanding social support among cadets.

**Figure 1:**
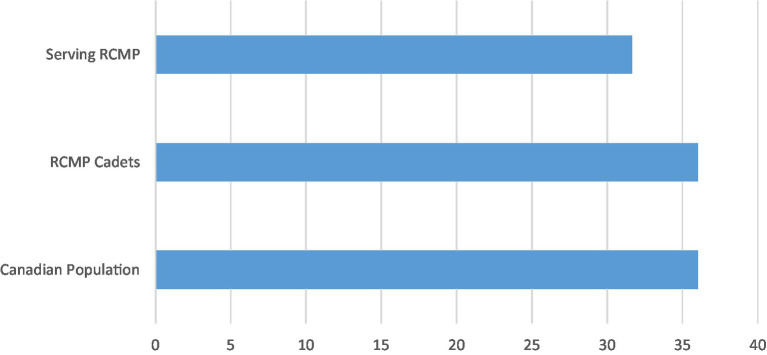
Total SPS-10 scores for the Canadian population, RCMP cadets, and serving RCMP.

Directional predictions were not made for the level of perceived social support reported by cadets when compared to the Canadian general population. Cadet scores were comparable to SPS-10 scores of the Canadian general population in 2012 (see Figure 1), with statistically significant lower scores reported by cadets for item four – (i.e., “I have close relationships that provide me with a sense of emotional security and well-being”) and statistically significant higher scores for item nine (i.e., “There are people who admire my talents and abilities”) in Table 3. The different collection periods may have impacted the levels of perceived social support reported. Further research is required to understand the nuances of these item-level differences.

Perceived social support was expected to be higher among cadets than among serving RCMP (Vig et al., 2020). Cadets reported statistically significantly higher perceived social support than serving RCMP based on both total SPS-10 scores and each item individually. The current results appear consistent with previous results wherein new firefighting recruits reported higher perceived social support earlier in the training process than active-duty firefighters, likely due to diminishing social support which may be related to years of service (Regehr et al., 2003) or perhaps fading public confidence and levels of trust in the organization (Adorjan et al., 2017; Government of Canada, RCMP, 2021). The longitudinal design of the RCMP Study will allow the research team to follow cadets and assess for changes in perceived social support, associations of such changes with other variables (e.g., changes in marital status, exposure to occupational stressors), and the impacts of changes in perceived social support on physical and mental health over time.

The results suggest that higher levels of perceived social support when starting the CTP are associated with lower odds of screening positive for PD, GAD, and SAD which is consistent with previous research (Dour et al., 2014). However, greater perceived levels of perceived social support were not associated with a decreased likelihood of screening positive for symptoms associated with PTSD or MDD (Vig et al., 2020). The relationship between perceived social support and AUD could not be assessed, as none of the cadets screened positive for AUD. Within the cadet specific population an AUD cut-point of 15 may be overly high and ongoing research on PSP recruits may consider utilizing a score of 8 to increase sensitivity in order to detect hazardous drinking (British Columbia Centre on Substance Use, 2019).

### Strengths and limitations

The current paper has several limitations that provide directions for future research. First, the voluntary nature of cadet participation created an unknowable influence from self-selection biases in the cadets who chose to participate. Second, the cross-sectional nature of the data preclude making any directional or causal conclusions. While these data are consistent with a potential causal or buffering role of social support on mental health challenges, it is also known that pre-existing mental health challenges may impact the extent of social support received (Thoits, 2011). Cadets who experienced a mental health challenge may have also experienced challenges with building and maintaining relationships that provide high levels of social support. Third, the most recent Canadian general population data collection on perceived social support used the SPS-5 for administrative reasons (Orpana et al., 2019); accordingly, there is a time-lapse gap and a reduction in the number of items which may preclude direct comparisons. Fourth, the Canadian general population sample is not matched with the cadets (e.g., cadets have a higher proportion of males than in the general population). A matched comparison sample might provide greater nuance in future studies. Fifth, the research team did not conduct a non-response analysis of cadets who chose not to participate in the study. Lastly, the screening measures for mental health disorders used in the current study are valid and reliable for use in clinical settings; nevertheless, diagnoses can only be made using clinical interviews. Several strengths offset the limitations of the current paper. First, the longitudinal design of the RCMP Study captures cadet data early in training, assessing pre-existing mental health and providing initial estimates of perceived levels of social support. Second, the current paper uses well-established and well-validated measures to collect data from a large sample of a novel population. Third, the current paper focuses on the individual level (i.e., cadets’ perceived social support) to provide clarity rather than the use of an institutional support measure (i.e., Institutional Support and Betrayal Questionnaire, Self-care and Mental Health Access for Public Safety) which may capture a limited understanding of social support when compared to the SPS-10. Future researchers may consider a mixed-methods study design that includes qualitative interviews or focus groups to better understand the social support construct from cadets’ perspectives and incorporating the voice of people with lived experience of the transition into CTP and into RCMP service. In future, the SPS-5 could be used instead of the SPS-10 to shorten the survey length and to allow direct comparisons with the most recent general population data on social support among the Canadian general population (Orpana et al., 2019).

## Conclusion

The current paper demonstrates high levels of perceived social support ubiquitous among cadets at the start of the CTP. Higher levels of perceived social support were inversely associated with positive screens for PD, GAD, and SAD suggesting that perceived social support may be a protective factor against the development of anxiety disorders, in particular. The current results are the first to evidence the stark contrast in perceived social support between new cadets and serving RCMP members, suggesting the possibility of substantial reductions in perceived social support as a function of service. The current results include initial estimates of perceived social support and the associations with mental health challenges prior to the onset of training, providing a valuable benchmark for future research with RCMP members, other police, and other PSP groups.

## Data availability statement

The datasets presented in this article are not readily available because the datasets will be made available only for independent confirmation purposes and only to persons with the necessary ethical and security clearances as defined by the research ethics board at the University of Regina and the contractual obligations with the Royal Canadian Mounted Police. Requests regarding the datasets can be made to the corresponding author.

## Ethics statement

Data for the current paper were collected as a part of the broader RCMP Study. The associated protocol paper provides full details of the RCMP longitudinal Study (Carleton et al., 2022). The RCMP Study was approved by the University of Regina Ethics Board on April 10, 2019 (File #2019-055), and the RCMP Research Ethics Board followed with approval on April 12, 2019 (File #SKM_C30818021312580). The study was also approved through a Privacy Impact Assessment as part of the overall National Administrative Records Management System approval (201611123286) and Public Services and Procurement Canada approval (201701491/M7594174191). The project is bound by the Privacy Act, R.S., 1985, c. P-21 and the Personal Information Protection and Electronic Documents Act, SC. 2000, c.5 and approved by Public Services and Procurement Canada (PSPC) M7594-171491/001/SS. The participants provided their electronically-recorded informed consent to participate in this study.

## Author contributions

All authors made substantial contributions consistent with the International Committee of Medical Journal Editors. Initial design for the current article was based on the following contributors, each of whom was responsible for overseeing their area-specific domains for assessment, all of whom reviewed, revised as necessary, and approved the final design in its entirety. JN, RNC, KA, and LJ: conceptualization. JN, LJ, and RNC: methodology. LJ, RNC, SS, and JN: validation. LJ, KM, JN, RNC, and SS: formal analysis. JN, LJ, KA, TT, RS, KM, RNC, and SS: investigation. RNC: resources, supervision, project administration, and funding acquisition. JN, LJ, KA, RS, and RNC: writing-original draft preparation. JN, LJ, KA, SS, RS, TT, KM, and RNC: writing-review and editing. All authors contributed to the article and approved the submitted version.

## Funding

RNC is supported by a Medavie Foundation Project Grant. SS is supported by a Tier 1 Canada Research Chair in Addictions and Mental Health. The current study was supported by the RCMP, the Government of Canada and the Ministry of Public Safety and Emergency Preparedness, and a Grant from the Medavie Foundation.

## Conflict of interest

The authors declare that the research was conducted in the absence of any commercial or financial relationships that could be construed as a potential conflict of interest.

## Publisher’s note

All claims expressed in this article are solely those of the authors and do not necessarily represent those of their affiliated organizations, or those of the publisher, the editors and the reviewers. Any product that may be evaluated in this article, or claim that may be made by its manufacturer, is not guaranteed or endorsed by the publisher.

## Footnotes

^1^*N.B*., The ethical approval and assessments of the RCMP Study precludes making de-identified data accessible.

^2^The SPS-10 data for serving RCMP is taken from the AX1 dataset, which was collected *via* an online survey for PSP during a five-month period from 2016 to 2017. The survey and data storage were approved by the University of Regina Institutional Research Ethics Board #2016–107. The SPS-10 specific data has not been published.
